# Salivary Biomarkers in the Control of Mosquito-Borne Diseases

**DOI:** 10.3390/insects6040961

**Published:** 2015-11-17

**Authors:** Souleymane Doucoure, Papa Makhtar Drame

**Affiliations:** 1 Institut de Recherche pour le Développement, Unité de Recherche sur les Maladies Infectieuses Tropicales Emergentes (URMITE) UM63: CNRS7278-IRD 198-INSERM U1095 Campus IRD-UCAD, BP 1386, Dakar 18524, Sénégal; 2 Laboratory of Parasitic Diseases, National Institute of Allergy and Infectious Diseases, National Institutes of Health, Bethesda, MD 20892, USA; E-Mail: papa.drame@nih.gov

**Keywords:** mosquito, control, exposure, salivary-proteins, biomarker

## Abstract

Vector control remains the most effective measure to prevent the transmission of mosquito-borne diseases. However, the classical entomo-parasitological methods used to evaluate the human exposure to mosquito bites and the effectiveness of control strategies are indirect, labor intensive, and lack sensitivity in low exposure/transmission areas. Therefore, they are limited in their accuracy and widespread use. Studying the human antibody response against the mosquito salivary proteins has provided new biomarkers for a direct and accurate evaluation of the human exposure to mosquito bites, at community and individual levels. In this review, we discuss the development, applications and limits of these biomarkers applied to *Aedes*- and *Anopheles*-borne diseases.

## 1. Background

The control of mosquito-borne diseases (MBDs) represents a major worldwide public health challenge, especially in tropical and subtropical regions. Only a few diseases essentially carry out the worldwide burden of MBDs. Among them, malaria—caused by *Plasmodium* protozoan parasites that infect the human host through the bite of female anopheline mosquitoes—was responsible for 207 million cases and 627,000 deaths in 2013 [1]. Dengue, caused by a flavivirus transmitted by the bite of infected female *Aedes* mosquitoes, is the most prevalent MBD in the world and currently affects more than 100 countries with up to 390 million cases annually [2]. It is estimated that MBDs, including Japanese encephalitis, West Nile virus, Chikungunya and lymphatic filariasis, represent 90% of the disability-adjusted life years caused by vector-borne diseases and take a dramatic toll on health and socioeconomic development in affected areas [3]. The high burden of MBDs is linked to a lack of effective treatment and an increasing resistance of pathogens and mosquito vectors to available drugs and insecticides, respectively [4]. In addition, there is no vaccine against the most prevalent MBDs; the only successful vaccine strategies that have been developed for humans are against yellow fever and Japanese encephalitis virus infections. Therefore, vector control is currently the most appropriate strategy to limit or stop the transmission of MBDs. For example, the control of *Aedes*- and *Anopheles*-borne diseases, the two main MBDs in terms of burden, is essentially based on insecticide indoor residual spraying (IRS), the use of insecticide-treated bed nets and larval control. These strategies aim to significantly reduce the density of the vector population, the level of the contact between mosquito vectors and human populations, and then the transmission of pathogens. Such interventions are associated with recent declines in MBD burdens across a range of settings over the world. However, the general burden of MBDs is increasing in several tropical countries primarily due to human travel, rapid urbanization and failures of preventative public health measures [5]. The evaluation of the risk of MBDs and the effectiveness of control programs is therefore a necessity for achieving the pre-elimination goals fixed for some diseases [6].

In this review, we highlight the current entomological and parasito-clinical methods that are routinely used to assess the level of exposure to *Anopheles* and *Aedes* bites and the effectiveness of control measures against these vectors. We then expose the new concept of “salivary biomarkers” (SBs) of mosquito bites and the impact of such SB tools on the assessment of risk of MBDs and the effectiveness of vector control measures in different settings. The effects of some epidemiological parameters (*i.e*., age, seasonality, differential use of vector control) on the reliability of these SBs as well as the limits of such method are also discussed in order to highlight current drawbacks in using SBs for operational research.

## 2. Assessment of the Risk of *Anopheles-* and *Aedes*-Borne Disease and the Effectiveness of Vector Control

The evaluation of the human exposure to vector bites, the risk of MBD transmission and the effectiveness of vector control strategies are routinely based on entomological methods and on parasito-clinical assessments. However, these methods are labor-intensive and difficult to sustain on large scales, especially when transmission and exposure levels are low during dry season, in high altitude, in urban settings, or after vector control). In addition, they give a measure at the community level rather than an individual assessment, which can be useful since heterogeneity in host exposure could have a significant impact on vector control effectiveness [6].

The entomological inoculation rate, the gold standard measure for *Plasmodium* transmission intensity to humans, is highly dependent on the density of human-biting *Anopheles* [7]. This density is estimated by trapping methods such as human-landing catches (HLC) of adult mosquitoes. HLC is commonly used for sampling host-seeking mosquitoes and for assessing the level of human exposure to *Anopheles* bites. However, the technique of HLC poses ethical concerns as the human “bait” could be exposed to malaria and other MBDs. In addition, this trapping technique is only applicable to human adults. It is difficult to extrapolate HLC results to children or to pregnant women who are the most vulnerable groups to malaria [8]. Mosquito larval and pupal stages are generally used to assess human exposure to *Aedes* bites. Several techniques including the Breteau index, the container index, the premise index, and the premise shading, are used to estimate the density of *Aedes* pupae and larvae. The counting of these aquatic stages gives an indirect estimation of the level of human exposure to adult *Aedes* bites. However, the mortality of the immature stages that influences the adult density can limit the accuracy of such estimations. In addition, large scale *Aedes* immature stages surveys are needed for a reliable assessment.

The entomological tools routinely used to estimate MBD transmission and the efficacy of vector control strategies can be complemented by parasitological and clinical data. However, these latter strategies could be subjected to variability between sites and may not be appropriate for early phase studies of vector control or for epidemic prediction [8]. Transmission estimates based on the prevalence or densities of human infection are susceptible to micro-heterogeneity caused by climatic factors and socioeconomic determinants of the host-seeking behavior. More recently, serological correlates of transmission intensity have been described; however, they represent long-term rather than short-term exposure data [9]. Therefore, they are not suitable to evaluate the short-term impact of vector control programs.

Altogether, evaluating the risk of MBDs and the effectiveness of vector control strategies using the current entomo-parasitological methods is challenging. Thus, the development of new tools to reliably assess human exposure to vector bites and monitoring changes over time at both population and individual levels have been prioritized. The use of an SB approach offers a certain improvement as it gives a direct measure of the level of exposure to vector bites. Indeed, an SB approach measures the markers that are specific to the contact between the vertebrate host and the invertebrate vector during the blood meal uptake.

## 3. Human/Mosquito Interactions during the Bite and Role of Mosquito Saliva

The bite of the mosquito for a blood meal is the key component of the interactions between the vector and the human. During the infection of the female mosquito, parasites or viruses progress from the blood meal to the midgut before reaching the salivary glands (SGs). From the SGs, they can be transmitted through subsequent bites of the female mosquito that needs to feed on vertebrate blood to gain nutriments required for the development and the maturation of its eggs. However, the proboscis of the vector is more than a simple syringe, allowing the mosquito to feed and to potentially transmit microbes to the vertebrates. Indeed, during the bites, the mosquito injects saliva, which has an important physiological action allowing the mosquito to safely take a blood meal. It is well known now that mosquito saliva injected in human skin during a blood meal contains a cocktail of bioactive molecules [10]. These molecules have substantial anti-hemostatic, anti-inflammatory, and immune modulatory activities that assist the mosquito in the blood-feeding process [11]. Some of these salivary compounds are essential to the pathogen life cycle [12] while the others may be useful as biomarkers of the risk of diseases.

## 4. Development and Evolutionof Salivary Biomarkers (SB)

The immunological interactions between the human and the mosquito vector have been studied in the aim to develop SBs of mosquito bites. This concept is based on the fact that mosquito saliva injected into the human host skin during the mosquito bite is immunogenic and can induce the production of specific human antibodies (Ab). The fundamental principle of the use of SBs is therefore simply to use mosquito salivary proteins to probe specific Abs in human sera. To this end, several immunological techniques have been developed. The enzyme linked immunosorbent assay (ELISA) represents the most widely used technique due to its relative simplicity and affordable cost. Indeed, the ELISA technique does not require sophisticated instrumentation and can give both qualitative and quantitative evaluation of the Ab anti-mosquito saliva. Two main steps are crucial in the development of an SB: the collections of human blood samples potentially containing anti-mosquito Abs and the production of salivary gland extracts (SGEs) which represent the source of antigens. Two major techniques have been used to collect human blood sample containing anti-saliva immunoglobulin (Ig). Plasma can be used to detect the presence of anti-saliva Ig. However, the use of dried blood spots on filter paper is now widespread as it avoids invasive blood collecting methods [13,14]. The use of filter paper allows a very easy conservation of the samples as the dried blood spots can be stored for a long time at 4 °C. The SGEs, which represent the source of antigens, are extremely fragile and relatively difficult to conserve. The first studies carried out on the development of SBs were based essentially on whole SGEs. However, SBs based on the use of whole SGEs may not be very reliable. Indeed, whole SGEs antigens do not give any indication of the protein and/or epitope that induces the Ab response. It is well known now that *An. gambiae* and *Ae. aegypti* mosquito saliva contain up to 15 and 10 salivary proteins, respectively [15]. Several *Aedes* and *Anopheles* salivary antigens have been shown to be very antigenic [16]. In addition, a number of mosquito salivary proteins are shared between species, genera and insect families. Therefore, the use of whole SGEs enhances the probability of anti-saliva Ab cross-reactivity that may impair the evaluation of the exposure to different vectors species within families or genera.

Recent progresses in sialotranscriptomic studies [17,18,19] have allowed the identification of more specific antigens that have enhanced the specificity of the SBs. Two main approaches have been used for that purpose: (i) the recombinant protein strategy based on the identification of genus-specific salivary proteins. Currently, two SBs, gSG6 and cE5 have been developed based on that approach [20]. The gSG6 and cE5 proteins represent good indicators of the human exposure to *Anopheles* mosquito bites. In addition to their immunogenicity, gSG6 and cE5 SBs are specific to anopheline mosquitoes. These two SBs present different features: gSG6 induces a short lived IgG4 response compared to a longer lived IgG1 response produced against the cE5 protein [21]. However, SB based on whole recombinant protein may contain more than one epitope. In addition, there may be other limitations linked to the complexity of the system used to produce a recombinant antigen; (ii) On the other hand, bioinformatic analysis of the sialotranscriptomic data has been used to design and then synthetize specific peptides. The advantage of this technique is that only one epitope is used to develop the SB evaluating the human exposure to mosquito bites. Currently, two SBs based on peptide design have emerged as good markers of exposure to *Aedes* and *Anopheles* bites. The gSG6-P1 and the Nterm-34 kDa peptides represent two powerful SBs evaluating the human exposure to *Anopheles* and *Aedes* bites, respectively [13,22].

Specific salivary recombinant proteins and peptides can be easily expressed in cell culture and by chemical synthesis, respectively. This contrasts with the production of whole SGEs that requires dissecting a large number of mosquitoes to have the optimal protein concentration for the immunological test. However, changes in the salivary proteome according to vector physiology or diet could affect the reliability of SGE-based SB. The change in salivary proteome could also result from SGE conservation, which is very challenging [23]. In fact, the sequence and feature of a peptide or protein used for SB are already known, and their purity and integrity could be checked after production. At present, there are several genus specific salivary biomarkers based on peptide [22] or protein [20] strategy. These biomarkers have evolved toward more genus than species-specific indicators [24]. The success of species-specific salivary biomarkers may need a more sophisticated approach. Nevertheless, the current SBs have been used to assess human exposure to *Anopheles* and *Aedes* mosquitoes and the effectiveness of vector control interventions as well as the risk of disease transmission.

## 5. Human IgG Responses against Mosquito Saliva: Biomarkers of the Exposure to *Aedes* and *Anopheles* Bites

The quantification of the specific Abs response to mosquito salivary proteins has allowed the development of biological markers for the assessment of individual exposure to the *Anopheles* and *Aedes* bites and thus to the risk of MBD transmission [25]. This property has been used to highlight the exposure to *Aedes* and *Anopheles* bites regardless the age of individuals [26]. The question is how anti-mosquito saliva Ab can be used as a direct measure of the human exposure level to *Anopheles* and *Aedes* vector bites. To answer that question, many studies on SB have used the environmental parameters that mostly determine the density and fluctuation of mosquito vectors. In mosquito endemic areas, rainfall intensity and the geographical situation can have a considerable impact on vector density and their heterogeneous distribution. It is well known that an increase in vector population is observed during the rainy season compared to the dry season. This feature has been used to test if the level of anti-saliva Ab can follow the level of exposure to vector bites.

**Figure 1:**
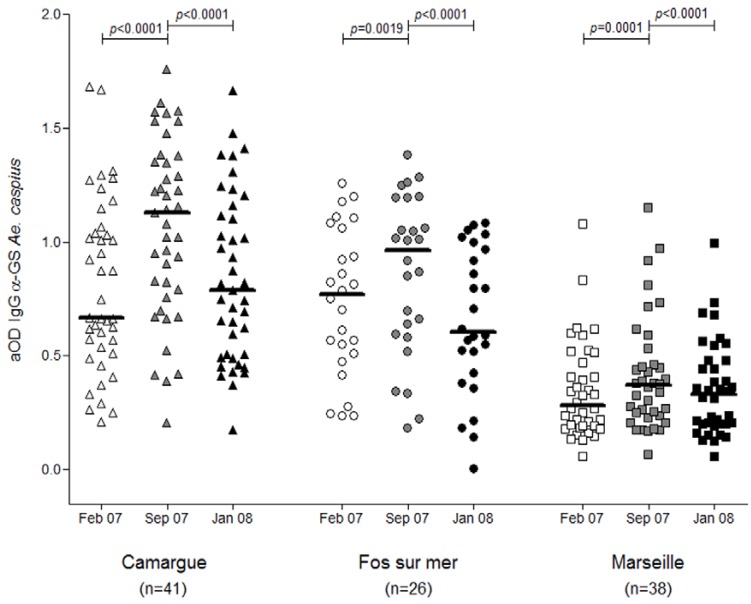
The fluctuation of the IgG anti-*Ae. caspius* saliva according the season of exposure. The measure of the IgG anti-*Ae. caspius* has been used to follow up the seasonal exposure to vector bites in three localities in Southern France. For each locality, the level of IgG against *Ae. cusp* saliva was measured in February (T1), September (T2) and January (T3) [28]. For each locality, the level of IgG against *Aedes caspius* saliva was measured in February (T1), September (T2) and January (T3), and is represented in white, grey and black symbols, respectively. Horizontal bars show medians.

Longitudinal studies conducted in a rural malaria endemic area in Burkina Faso have shown that the anti-gSG6 IgG could give an indication of the seasonal fluctuation of vector density. The use of indoor pyrethrum catch has shown that the exposure to *Anopheles* bites is higher during the rainy season periods. The individual levels of anti-gSG6 IgG are higher during the peak of exposure to *Anopheles* bites in the rainy season compared to the dry season. Furthermore, the prevalence of immune responders decreased during the dry season to less than 60% while it ranged between 60% and 75% during the rainy seasons periods [27]. The same observation was made with *Ae. caspius* in Southern France [28]. Despite the lack of entomological data, the anti-*Ae. caspius* SGE IgG followed the seasonal fluctuation of this mosquito species during a season of low exposure (T1), followed by the peak (T2) and a second period of low exposure (T3). The SB indicated an increase of immune responders going from 29% to 54% at T1 and T2, respectively. This study emphasized also the fact that the seroprevalence decreased after the peak of exposure at T3 and returned to the baseline level at 29% [28] (Figure 1).

In a cohort of Senegalese children, the level of IgG4 specific to *Ae. aegypti* saliva increased during the rainy season corresponding to a high proliferation of *Aedes* vectors with up to 56.25% of the human population developing a specific IgG4 response. In contrast, during the dry season, water sources were rare and then the *Aedes* population decreased as well as the level of human exposure to bites. The prevalence of immune responders decreased to 47% during this period of vector scarcity [29]. In Benin, a seasonal increase of the level of Ab according to the vector density has been confirmed during a two year longitudinal survey in the rainy and dry seasons. The use of a specific peptide helped to show that the level of anti-Nterm-34 kDa IgG followed the rainfall intensity. The level of immune responders varied from 63.42% during the dry season to 97.28% in the rainy season [22]. The seasonal increase associated with the rainfall intensity is not limited in tropical areas—the same trend was observed in the Finnish population with an increase of the level of IgE and IgG4 specific to *Ae. communis* saliva [30].

The distribution of vector populations according to different geographical features also helped to validate the use of the SB approach as an indicator of exposure to *Aedes* and *Anopheles* mosquitoes. In Southern France, the spatial distribution of *Ae. caspius* was heterogenous and depended on the ecological environment. In a wetland area situated in the Rhone River delta, near Camargue, *Ae. caspius* densities were very high whereas these densities were very low in the urbanized city of Marseille. Intermediate *Ae. caspius* densities were described in the city of Fos-sur-mer, which is located between the Camargue area and Marseille. The differences of exposure occurring between these three ecological environments have also been described by measuring the level of anti-*Ae. caspius* SGEs IgG. The level of IgG and the prevalence of IgG responders followed the distribution of vector density in these three areas regardless the season of exposure—for example, during the peak of the exposure, the prevalence of IgG responder was higher in Camargue (55%) than in Fos-sur-mer (40%) [28].

**Figure 2:**
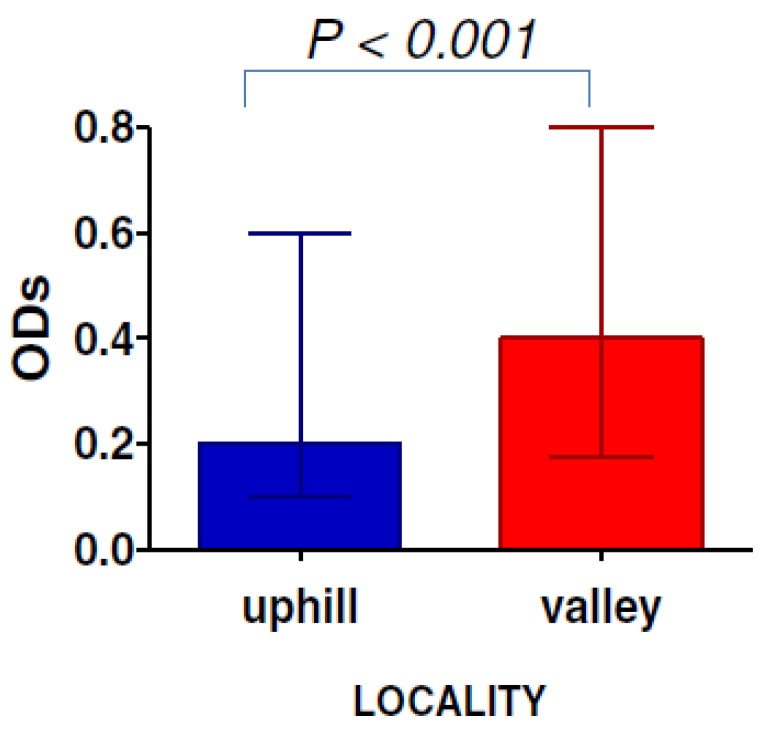
The gSG6-P1 SB indicating the exposure to *Anopheles* in two different geographical localities. The gSG6-P1 could be used to highlight the difference of exposure to *Anopheles* bites in two geographical settings marked by different vector densities. The optical density values (ODs) on the y axis of the graph represent the level of IgG response to gSG6-P1 SB [31].

The density of some vectors varies according to altitude. It is well known that *Anopheles* density is lower at higher altitude compared to lower altitude areas. In Kenyan highland areas, the use of SB gSG6-P1 showed a difference of exposure between populations residing in “uphill” to those living in “valley bottom” areas. Indeed, the median IgG level was twice as high in the valley population than in the “uphill” population. Similarly, the prevalence of the immune responders was 36% and 50% in “uphill” and bottom valley areas, respectively [31] (Figure 2). Therefore, the use of the gSG6-P1 SB showed changes in mosquito exposure that could be expected from a difference on mosquito density driven by the altitude.

The ability to use the human host Ab response as an indicator of the exposure to vector bites relies on the fact that such Ab response is stimulated by the exposure to vector bite and this response does not build up. It wanes rapidly without repetitive exposure to vector bites. This has been shown principally with total short-lived IgG. In adult individuals, the proportion of immune responders can decrease from 100% to 67% and to 47% within two and six weeks, respectively, when exposure to vector bites is not sustained [32]. In the field, where individuals are continuously exposed to arthropods bites, it is difficult to determine the time it takes to develop an anti-saliva Ab response after the exposure to mosquito bites. Some studies have revealed that it can take one to two months. However, the appearance of the anti-vector salivary proteins Ab can be as quick as one week in some experimental models [33]. More than detecting the fluctuation of the level of anti-salivary proteins Ab, the SB should meet strict criteria of sensitivity and specificity to accurately measure the human exposure to vector bites. To this end, it has been shown that SBs provide sensitive detection of the level of exposure to *Ae. aegypti* bites, as estimated by other standard entomological techniques [34]. It also has been shown that SBs give a sensitive measure of low level exposure to anopheles bites [35]. This high sensitivity of SBs helps detect the heterogeneity of exposure to vector bites that can also occur in the context of low level density of *Anopheles* population [36]. For the relevancy of SB, this sensitivity has to be accompanied by a high specificity. Most notably, unexposed individuals should not be able to develop a specific Ab response against mosquito saliva [37]. A low level of anti-saliva IgG cross reactivity was found between two populations exposed to bites of *Ae. albopictus* or *Ae. aegypti* [37]. These different findings show that the SBs are highly sensitive for the detection of human exposure to mosquito bites, the fluctuation of vector density depending on ecological parameters. These features make the SB of considerable value for the evaluation of the efficacy of vector control interventions and the risk of MBDs.

## 6. Mosquito Salivary Biomarkers for Monitoring Vector Control Strategies

**Figure 3:**
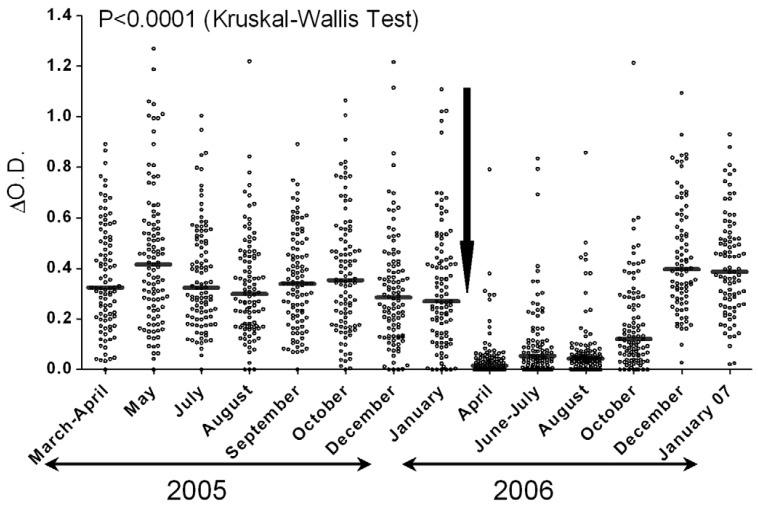
The IgG anti- gSG6-P1 evaluating the efficacy of LLIN use. The gSG6-P1 SB represent a good indicator for LLIN efficacy and their non-use or damage over time [38].

SBs have been also used to monitor the efficacy of vector control targeting *Aedes* and *Anopheles* mosquitoes. Monitoring the efficacy of anti-malaria vector strategies is challenging. Large parasitological surveys are needed to evaluate the impact of anti-malaria vector control strategies based on long lasting insecticide nets (LLINs). In this context, the use of SB could be a complementary tool. Indeed, in Angola, it has been proven that the efficacy of LLINs use is accompanied by a decrease of the level of exposure to *Anopheles* bites and/or the prevalence of *Plasmodium* within a population [14]. The decrease of these two parameters has been observed in a population of adults and children after the implementation of LLINs [14]. The period of decrease of the level of exposure to mosquito bites and the prevalence of *Plasmodium* coincided with the collapse of both the intensity of anti-*An. gambiae* saliva IgG and the prevalence of immune responders measured before and after the implementation of LLINs [14]. These results, therefore, provide a clear indication of the possibility to use SBs for the evaluation of the efficacy of vector control strategies. Moreover, the use of the specific salivary peptide gSG6-P1 has enabled the collection of relevant data on the efficacy of LLINs over time. It was possible to detect an increase in the level of exposure caused by the LLINs damage or non-use. In the same study, the authors have shown that gSG6-P1 SB is a good marker of the short term efficacy of LLINs use whereas no significant change in the density of *Anopheles* mosquitoes, measured by classical entomological methods, was observed [38] (Figure 3). Similarly, the measure of the IgG response helped in assessing the efficacy of control strategies based on deltamethrin spray and physical elimination of breeding sites two weeks after the implementation of these control measures. This result is particularly important for the relevancy of SBs in that no significant decrease of the Breteau index (BI) and adult density was observed using the standard entomological techniques [39]. These studies show that the SB is a pertinent tool for assessing the efficacy of different vector control strategies over a short period. Furthermore, it has been used to compare the efficacy of concomitant mosquito control strategies and indicated that the combination of two vector control methods is more effective than one control method only [40]. It has provided the first opportunity to compare the efficacy of individual protection tools such as insecticide spray bombs and mosquito coils to LLINs, indicating that the decrease in *Anopheles* aggressiveness has essentially been due to the use of nets [13].

## 7. The Salivary Biomarkers and the Risk of Mosquito Borne-Diseases Transmission

The evaluation of the risk of disease transmission is an essential component of current entomological techniques. The use of SBs should meet this criterion in order to be considered as a complementary disease diagnostic tool. Early studies have clearly linked the anti-saliva IgG response to the risk of disease transmission. It has been observed that the level of anti-mosquito saliva Ab is higher in infected individuals compared to healthy subjects. In seasonal and moderate malaria transmission areas in Africa, the level of anti-*An. gambiae* saliva IgG was significantly higher in children with malaria compared to uninfected children [25]. The same trend was also observed in adults living in an area of intense malaria transmission in Thailand. In addition to having the highest anti-saliva Ab, the levels of anti-*An. dirus* SGE IgG were more heterogenous in malaria patients compared to healthy people. However, the use of the SBs failed to show a correlation between the anti-*Plasmodium* sporozoite Ab and the anti-*An. dirus* SGEs IgG. This study revealed also that the level of anti-*An. dirus* SGEs IgM was significantly elevated in infected individuals [41]. However, in Haitian people, no significant difference in the level of IgM Ab against *An. albimanus* was found between uninfected and malaria infected individuals in spite of a higher level of anti-vector saliva IgG in this last group [42].

**Figure 4:**
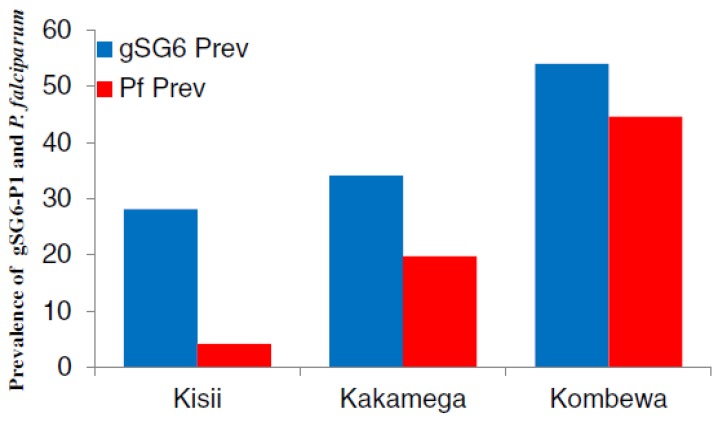
The gSG6-P1 and malaria transmission. The SB showed that the malaria prevalence in Kisii (*n* = 222), Kakamega (*n* = 203) and Kombewa (*n* = 202) depends on the level of exposure to vector bites [31].

In contrast to IgM, IgG Abs are often used to evaluate the risk of malaria transmission and this Ab isotype has given reliable indication on the capacity of both the gSG6-P1 and gSG6 SBs to highlight malaria infection in endemic areas. For example, in Kenya, the level of anti-gSG6-P1 IgG varied according to malaria transmission intensity. Indeed, the percentage of anti-gSG6-P1 IgG responders was 28% in a hypoendemic region, 34% in a mesoendemic region, and 54% in a hyperendemic region, where the parasite prevalences were shown to be 4%, 19.7% and 44.6%, respectively. Furthermore, the risk of seroconversion to Merozoite Surface Protein1-19 (MSP-1_19_) was three times higher in anti-gSG6-P1 IgG positive individuals than negative ones [31] (Figure 4). These results indicated the accuracy of the SB for assessing the risk of disease transmission as MSP antigen represents a benchmark for the evaluation of malaria transmission intensity. This relationship between the SB and markers of malaria exposure was confirmed in an area of moderate malaria transmission in Tanzania where the incidence of malaria was significantly associated with the prevalence of anti-gSG6, MSP-1 and GLURP R2 IgG [43]. Remarkably, the gSG6-P1 has been used as a biomarker of infection in a low malaria transmission area and in the particular context of the dry season when malaria cases are very rare. This study revealed that the levels of anti-gSG6-P1 IgG are significantly higher in infected children compared to healthy ones both at the beginning and at the end of the dry season. Furthermore, this study showed that the anti-gSG6-P1IgG could be used to distinguish healthy individuals from asymptomatic *Plasmodium*-infected children and clinical malaria cases. Indeed, the level of anti-gSG6-P1 is low, moderate and high in uninfected-, infected-asymptomatic and infected-symptomatic individuals, respectively [36]. However, this is contrary to an investigation conducted in the Brazilian Amazon where the level of anti-*An. darlingi* saliva IgG is higher in asymptomatic *P. vivax* carriers than symptomatic patients [44].

Further studies are needed to clearly define the link between the level of IgG Ab against vector salivary proteins and malaria outcomes. This could be of particular importance when it is known that the antigenicity of *Ae. aegypti* salivary proteins may depend on the severity of dengue disease [45]. However this does not prevent the use the IgG Ab as the optimal isotype for assessing the risk of mosquito borne-diseases transmission as confirmed by the Dengue disease model. Indeed, significantly higher anti-vector saliva IgG was observed in dengue febrile individuals compared to uninfected individuals [32]. The use of the Nterm-34 kDa peptide SB could not distinguish a difference between Dengue positive and Dengue negative individuals; however, it was used to retrospectively identify urban areas of Vientiane, Laos that were at higher risk of Dengue virus transmission [46]. These studies therefore suggest that, in endemic areas, the level of IgG against vector saliva could represent a genuine biomarker to identify the risk of MBD transmission.

## 8. The Limits of Current Salivary Biomarkers

The pertinence and the large-scale application of SB for epidemiological purposes have been hampered by several limitations. First, the whole saliva of mosquitoes is a cocktail of various molecular components with different chemical natures and biological functions. Some components are *Aedes* or *Anopheles* specific and other are widely distributed within genera, orders or classes of blood sucking *Diptera*. Therefore, the evaluation of *Anopheles/Aedes* exposure or vector control effectiveness based on the immunogenicity of whole SGEs could be skewed and over- or under-estimated by possible cross-reactivity between common epitopes between mosquito species or other organisms [20]. Second, the collection of saliva or salivary gland extracts is tedious and time consuming; therefore, it will be difficult or impossible to have an adequate production of mosquito saliva needed for large-scale epidemiological studies. Third, saliva composition can be affected by several ecological parameters such as the age, physiological status or infection of the vector [11], which may influence the anti-saliva immune response measured and therefore result in a lack of reproducibility between saliva batches. The recent use of species/genus-specific proteins as an alternative for optimizing the specificity of such immunological biomarkers has also shown some limitations. Indeed, the gSG6-P1 assesses the level of exposure to both infected and uninfected *Anopheles* bites. In hyperendemic malaria areas, residents are also highly exposed to non infectious bites. Thus, they present very high levels of anti-gSG6-P1 Ab, which does not necessarily reflect the intensity of malaria transmission. Therefore, there is a need for biomarkers of exposure specific to infective bites in order to assess directly the risk of malaria transmission. Indeed, the tests for pathogen proteins within human are currently used in estimating the risk and prevalence of disease transmission. However, in humans, this estimation may not provide a true indication of transmission risk as some people are asymptomatic pathogen carriers and have developed a relative immunological protection against the disease. An SB of infective bites would help to identify individuals who have been in contact with an infected vector and therefore would help to more accurately evaluate the risk of disease transmission. Its combination with the current test for pathogen detection would be beneficial in the context of the latter stages of a malaria pre-elimination strategy when transmission levels would be very low. Recent studies demonstrate the feasibility of an SB of infective bites as the amount of some antigenic salivary proteins is significantly changed with vector infection [12]. Another limitation of the current SB strategy is the current level of development and applicability in the context of operational research and mosquito control interventions. For most of the current entomological measurement techniques, the application process is well defined and is achievable by non-specialized field workers. Currently, specially trained staff is needed to carry out the immunological tests required for the detection of Ab or to dissect mosquito salivary glands. Additional work is then needed to refine strategies of measurement with the SBs by non-specialized workers. Therefore, it would be beneficial to develop rapid diagnostic tests for the measurement of SBs to enable its widespread use.

## 9. Conclusions

Salivary biomarkers based on the human Ab reaction against mosquito salivary proteins could represent complementary tools to the current entomological techniques. The SBs could be used for a broad range of populations of different ages and in different contexts of MBD transmission. Their use could help to improve the evaluation of vector control interventions and the risk of disease transmission. Their high sensitivity constitutes an input for the current mosquito control strategies particularly when vector density is low as in areas where malaria pre-elimination programs are implemented. However, further improvements are needed to overcome some constraints related to their use.
